# Oral Complications related to tropical infectious Diseases: an introduction and analysis of survey data

**DOI:** 10.1186/s12903-023-03514-w

**Published:** 2023-10-24

**Authors:** Yi Shuai, Yanfeng Lou, Lei Zhu, Wei Chen, Tao Jiang

**Affiliations:** 1 Department of Stomatology, Jinling Hospital, Medical School of Nanjing University, Nanjing, 210002 Jiangsu China; 2 Department of Stomatology, General Hospital of Eastern Theater Command, PLA, Nanjing, 210002 Jiangsu China

**Keywords:** Tropical infectious Diseases, Oral health, Infectious Disease practitioners, Understanding

## Abstract

**Backgrounds:**

The non-indigenous tropical infectious diseases that occur in the non-tropics arise from personnel communication between locals and visitors. Many of these infectious diseases involve oral complications. It is very important for practitioners to manage such cases based on a clear understanding of the association between tropical infectious diseases and oral health. In this study, we summarize the oral complications related to tropical infectious diseases and investigate the understanding of infectious disease practitioners in relation to the association between these conditions. In addition, we provide supportive advice to facilitate the oral management of tropical infectious diseases.

**Methods:**

First, we investigate the oral complications related to tropical infectious diseases by performing an appropriate literature search. Then, we analyzed the understanding of 207 Chinese practitioners specializing in infectious diseases in relation to the association between these two conditions by applying a bespoke online questionnaire.

**Results:**

Analysis revealed that the Chinese practitioners had very poor attitudes and knowledge relating to the association between tropical infectious diseases and oral health. Different backgrounds had no significant impact on the understanding of Chinese practitioners with regards to the association between tropical infectious diseases and oral health.

**Conclusion:**

Many oral complications are related to tropical infectious diseases. The understanding of Chinese practitioners with regards to the association between infectious disease and oral health was very inadequate. It is essential to promote publicity and education relating to infectious tropical diseases and oral health.

**Supplementary Information:**

The online version contains supplementary material available at 10.1186/s12903-023-03514-w.

## Background

According to data published by the World Health Organization (WHO), millions of cases involving tropical infectious diseases occur in tropical regions; some of these can be disabling and even life-threatening [1]. Infectious diseases are highly prevalent in tropical areas. However, an increase in international travel and personal communication between visitors and locals can lead to local outbreaks [2–4] or widespread epidemics of non-indigenous diseases in non-tropical regions [5–7]. Therefore, tropical infectious diseases are gradually becoming a global public health concern. One of the key goals of the ‘WHO African Region Communicable Diseases Cluster Annual Report 2016’, was to reduce the incidence and burden of infectious diseases, and a team of public health experts were tasked to accomplish this goal [1]. Infectious disease practitioners, a crucial group of public health experts, are regularly challenged by tropical infectious diseases and are expected to control such problems. The attitude and knowledge of infectious disease practitioners play a critical role in the management of infectious diseases.

The oral-maxillofacial region is often subject to attack by infectious diseases [8]. The oral complications related to tropical infectious diseases have been investigated previously (Table 1) and parasitic diseases (malaria, leishmaniasis, and amoebiasis) [8–16], bacterial diseases (leprosy, and yaws) [8, 17, 18], viral diseases (dengue fever and measles) [8, 16, 19–21] and fungal diseases (paracoccidioidomycosis and histoplasmosis) [8, 18, 22–26], such as oral mucosal hemorrhage, oral mucositis, oral ulceration, enamel hypoplasia, and alveolar bone disorders [8, 15, 16, 23]. In addition, the current medications that are available for the treatment of tropical infectious are also associated with some oral adverse events (Table 1), including pain in the oral soft tissue, toothache, enamel hypoplasia, periodontal diseases, and stomatitis [8, 15, 27, 28]. To provide convenient medical guidelines, we have summarized various oral complications related to tropical infectious diseases, particularly associated with the oral-maxillofacial region (Table 1). Some oral complications are acute, while others are long-term (Table 1). Moreover, some cases of infection can present with oral manifestations as the first symptoms [15, 29–31]; these symptoms can easily be overlooked by clinicians. Infectious disease practitioners and dentists are the most relevant professions to manage and treat oral complications caused by tropical infectious diseases. In a previous study, we reported that the Chinese dental professionals had poor attitudes and knowledge relating to oral health and tropical infectious diseases [8]. Our findings clearly highlighted that infectious disease practitioners should pay more attention to the oral complications related to tropical infectious diseases to prevent misdiagnosis and epidemic spread. Differential diagnosis and appropriate treatments might improve the clinical management of tropical infectious diseases, which primarily depends on basic education and continuing clinical education. Nevertheless, few studies have investigated this issue with regards to the association between tropical infectious diseases and oral health.

**Table 1:** Oral complications related to tropical infectious diseases

Tropical Infectious Diseases	Relevant oral complications	
	Acute phase changes	Long-term changes
Parasitic diseases		
Malaria	Gingival bleeding; glossitis; oral ulcer; herpes labialis; herpes gingivostomatitis; pericoronitis; bitter taste; sore throat	Alveolar bone resorption; burkitt lymphoma of the jaw; enamel hypoplasias; oral pigment
Leishmaniasis	Oral pain	Destructive granulomatous lesions of facial skin, lips, buccal mucosa, palate and tongue
Amoebasis	Moist tongue; furred tongue	N/A
Trypanosomiasis	Myoclonus of lips	Lipochagomata genii (painful, ovoid, purple patches of buccal bilateral fat pad)
Ancylostomiasis	Extreme pallor of oral mucosa and lips; glossodynia; angular cheilosis	Atrophy of lingual papilla
Trichuriasis	Ulcerative stomatitis; glossitis	Hyperplastic gingivitis
Trichinosis	Recurrent mandibular swelling with pain; dry mouth; oral ulceration; facial myalgia	Diffuse indefinite radiolucency on the alveolar crest; hyperplastic gingivitis; difficulty in mastication, deglutition and speech; associated with oral squamous cell carcinoma
Filariasis	Oral manifestations mainly occur in female sufferers; asymptomatic, solitary swelling of lips, tongue, gingival papillae and buccal mucosa	Asymptomatic, solitary swelling of lips, tongue, gingival papillae and buccal mucosa
Taeniasis	Ulcerative, hemorrhagic stomatitis, gingivitis and stomadynia	N/A
Echinococcosis	N/A	Painless, solitary, firm or fluctuant swelling of hydatid cysts in major salivary glands, jaw bones, tongue and buccal mucosa
Cysticercosis	N/A	Cystic nodules on the tongue, buccal mucosa, lips and facial skin
Sparganosis	N/A	Asymptomatic submandibular or labial mass
Bacterial diseases		
Leprosy	Hemorrhagic sessile nodules, necrosis, fibrosis and ulcer of oral mucosa; fissured tongue; lepromata on the tongue; atrophic papillae and loss of taste; smokers palate; gingivitis; oral candidiasis; erythematous infiltrated plaque of facial part	Loosening, dysplasia and dental pulp necrosis of maxillary anterior teeth; periodontitis; oral melanosis; oral depigmentation
Yaws	Gangosa (destruction of hard palate and maxillary nasal processes)	Gangosa (with heavy scarring)
Viral diseases		
Dengue fever	Acute hemorrhage of gingiva, palate and tongue; blisters of tongue and palate; erythematous plaque of oral mucosa; dry mouth; oral candidiasis; swallowing difficulty	Osteonecrosis of the jaw; taste changes; post-extraction hemorrhage
Measles	Facial rashes; Koplik’s spots on oral mucosa; necrotic stomatitis; oral ulcer; pericoronitis; gingivitis; oral candidiasis	Koplik’s spots on oral mucosa
Fungal diseases		
Paracoccidioidomycosis	Painful proliferative erythematous granulomata of tongue, lips, gingiva, palate, alveolar ridge, pharynx, labial and buccal mucosa with ulceration, gingival bleeding; loosening of teeth; pigmentation of oral mucosa; fibrosis and cicatrization; facial lymphadenopathy; sialorrhea	Loosening of teeth; pigmentation of oral mucosa; fibrosis and cicatrization; facial lymphadenopathy
Histoplasmosis	Granulomas or fungating ulcers on lips, buccal mucosa, tongue, palate, gingiva and alveolar ridge with an indurated border; sore pharynx and larynx;	Dysphonia; dysphagia
Rhinosporidiosis	N/A	Exuberant granulomatous lesions of jawbone, tongue, soft palate or oropharynx
Oral adverse events asscociated to the medications for tropical infectious diseases		
Antimalarials	Oral soft tissue pain; toothache; oral paresthesia; oral hemorrhage; oral ulceration; stomatitis; simple cutaneous and mucosal lesions; herpes labialis; facial herpes zoster; tongue disorders or symptoms; tonsillitis; bitter taste; halitosis; facial swelling; facial skin lesions of severe system adverse events; sore throat	Enamel hypoplasia; tooth discoloration; oral lichenoid reaction; oral hyperpigmentation; periodontal diseases; salivary gland disorders

Therefore, it is essential to investigate the oral complications related to tropical infectious diseases and determine the understanding of the infectious disease practitioners with regards to tropical infectious diseases and oral health, and to identify the potential influence of different backgrounds and experiences on clinical outcomes. We hope that our findings will serve as a warning to practitioners facing tropical infectious diseases to improve their understanding of the management of tropical infectious diseases and oral health.

## Methods

### Ethics

The study was approved by The Institutional Review Board/Ethics Committee of Jinling Hospital. Informed consent was obtained in the online questionnaire completed by all participants. The study protocol conformed strictly to the tenets of the Declaration of Helsinki.

### Questionnaire preparation

The online questionnaire (The Tencent Technology Co. Ltd, China) was designed by three professionals with experience in infectious diseases, stomatology and tropical medicine, and was validated by a pilot study of 16 participants from Jinling Hospital, Medical School of Nanjing University. The questionnaire was modified according to feedback provided by the participants and the suggestions of experts. The Cronbach’s α Coefficient (0.90) and Goodness of Fit Index (0.81) were applied to estimate the reliability and validity of the questionnaire by statisticians. The final questionnaire (see Supplementary Data) featured 36 questions, including individual information (six items, multiple choice, description), attitude towards oral complications related to tropical infectious diseases (five items, yes/no), the understanding of oral complications related to tropical infectious diseases (21 items, multiple choice), and the management of oral complications related to tropical infectious diseases (four items, multiple choice). Answers were evaluated in accordance with the standard answers we provided. The methodology used was based on our previous research [8].

### Study participants and analysis

According to the survey, by 2021, there were 98,978 registered infectious disease practitioners in China [32]. The sample size was calculated with a confidence level of 95%, an error of 10% and a population percentage of 50%. The calculated sample size was 97. In total, 213 infectious disease practitioners from 12 provincial regions of China responded to the questionnaire. The respondents participating in the pilot study were excluded from the final study. The participants of the final study were recruited from 12 provincial regions by online recruitment *via* the China Infectious Disease Professional Society. The completed questionnaires were received from the 18th of March 2022 to the 25th of March 2022; 207 questionnaires were valid. The final data were statistically analyzed with regards to four different backgrounds and experience. The total score of each part was normalized to 10, as described in our previous study [8].

### Statistical methods

Descriptive statistics were applied for absolute numbers and percentages to evaluate information relating to the understanding and personal attitudes of each participant. Scores are described by mean ± standard deviation (SD) and quartiles. The Chi-squared test and continuity correction was applied to estimate ratios among different groups. The Student’s t test and one-way analysis of variance (ANOVA) were applied to compare means between different groups. *p* < 0.05 was regarded as statistically significant. Our statistical approach was based on our previous research [8].

## Results

### Sample characteristics

A total of 213 infectious disease practitioners from 12 provincial regions of China were recruited as participants. Of these, 207 participants completed the questionnaire (97.18%). Of the 207 participants, 109 were male and 98 were female; 141 were postgraduates, 42 were graduates and 24 were junior college graduates. The participants included 177 doctors and 30 nurses; 21 of whom had senior titles and 120 had intermediate titles. Sixty-six participants had junior titles; 25 had working experience in the tropics while 182 participants did not. The mean age of the participants was 36.2 year-of-age (range: 24–57 years-of-age) (Table 2).

**Table 2:** Sample characteristics

Category	Composition
Sample size	207
Gender	Male (109), Female (98)
Age	Average 36.2 ± 7.1 Y
Education background	Postgraduate (141), Graduate (42), Junior college diploma (24)
Professional identity	Doctor (177), Nurse (30)
Professional title	Senior (21), Intermediate (120), Junior (66)
Tropics working experience	Yes (25), No (182)

Note: Professional title: Senior (chief physician, associate chief physician, chief superintendent nurse, associate chief superintendent nurse), Intermediate (attending physician, nurse-in-charge), Junior (resident physician, primary nurse, nurse)

### The attitude of the Infectious Disease practitioners towards tropical infectious Diseases and oral health

Next, we investigated the oral complications related to tropical infectious diseases. The proportion of positive attention and systematical study experience of the infectious disease practitioners with regards to tropical infectious diseases and oral health were only 20.8% and 24.6%, respectively (Table 3). The proportion of participants who thought it was necessary to systematically learn about infectious diseases and oral health if working in non-tropical regions was 41.1%; this proportion was significantly higher in those participants working in the tropics (51.2%; Table 3). The proportion of participants with regards to the identification of oral lesions being helpful in the management of tropical infectious diseases was 35.7% (Table 3).

**Table 3:** The attitude of the infectious disease practitioners towards tropical infectious diseases and oral health

Content of questionnaire	Total(207)	Education background				Professional identity			Professional title				Tropics working experience		
	**positive**	postgraduate (141)	graduate (42)	junior college diploma (24)	*p*	doctor (177)	nurse (30)	*p*	senior (21)	intermediate (120)	junior (66)	*p*	Yes (25)	No (182)	*p*
Focus on tropical infectious diseases and oral health.	**43** **20.8%**	30 21.3%	9 21.4%	4 16.7%	0.870	38 21.5%	5 16.7%	0.549	7 33.3%	28 23.3%	8 12.1%	0.064	6 24.0%	37 20.3%	0.671
Study on tropical infectious diseases and oral health systematically.	**51** **24.6%**	38 27.0%	10 23.8%	3 12.5%	0.313	47 26.6%	4 13.3%	0.185	8 38.1%	32 26.7%	11 16.7%	0.102	8 32.0%	43 23.6%	0.362
If working in non-tropics, it is necessary to learn about infectious diseases and oral health systematically. ^§^	**85** ^**§**^ **41.1%**	57 40.4%	18 42.9%	10 41.7%	0.959	76 42.9%	9 30.0%	0.183	9 42.9%	49 40.8%	28 42.4%	0.970	11 44.0%	74 40.7%	0.750
If working in tropics, it is necessary to learn about infectious diseases and oral health systematically. ^#^	**106** ^**#**^ **51.2%**	71 50.4%	22 52.4%	13 54.2%	0.929	92 52.0%	14 46.7%	0.590	11 52.4%	65 54.2%	30 45.5%	0.520	15 60.0%	91 50.0%	0.348
Identification of oral lesions is helpful to the management of tropical infectious diseases.	**74** **35.7%**	51 36.2%	15 35.7%	8 33.3%	0.965	64 36.2%	10 33.3%	0.765	8 38.1%	42 35.0%	24 36.4%	0.956	9 36.0%	65 35.7%	0.978

**Note**: Item “#” compared with item “§”, *p* = 0.107. Professional title: Senior (chief physician, associate chief physician, chief superintendent nurse, associate chief superintendent nurse), Intermediate (attending physician, nurse-in-charge), Junior (resident physician, primary nurse, nurse)

There was no significant difference between participants with no experience of working in the tropics when compared to those with such experience (Table 3). In addition, there was no difference between participants with different educational backgrounds, professional identities, and professional titles (Table 3).

### The knowledge of the Infectious Disease practitioners on tropical infectious Diseases and oral health

Mean scores relating to the knowledge of oral complications related to tropical infectious diseases and the adverse oral events caused by medications for tropical infectious diseases were only 2.32 ± 1.28 and 1.02 ± 0.79, respectively (Table 4). The 1/4 percentile scores corresponding to such knowledge did not exceed 2.00, the median score did not exceed 2.00, and the 3/4 percentile score did not exceed 4.00 (Table 4). The mean score of oral complications related to tropical infectious diseases showed statistical differences when compared across different professional identities (doctors and nurses; (2.44 ± 1.26 vs. 1.63 ± 1.59, respectively; Table 4). However, there was no significant difference of these scores when compared between different educational backgrounds, professional identities, professional titles and working experience in the tropics (Table 4).

**Table 4:** The knowledge of the infectious disease practitioners towards tropical infectious diseases and oral health

Content of questionnaire			The knowledge towards tropical infectious diseases and oral health	
			oral complications related to tropical infectious diseases	oral adverse events of the medications for tropical infectious diseases
**Total score**		**average**	**2.32 ± 1.28**	**1.02 ± 0.79**
		**1/4**	**2.00**	**0.00**
		**1/2**	**2.00**	**1.00**
		**3/4**	**3.00**	**2.00**
Education background	postgraduate	average	2.32 ± 1.15	1.02 ± 0.79
		1/4	2.00	0.00
		1/2	2.00	1.00
		3/4	3.00	2.00
	graduate	average	2.50 ± 1.56	1.02 ± 0.80
		1/4	1.25	0.00
		1/2	2.00	1.00
		3/4	4.00	2.00
	junior	average	2.00 ± 1.35	1.04 ± 0.79
		1/4	1.00	0.00
		1/2	2.00	1.00
		3/4	3.00	2.00
Professional identity	doctor	average	2.44 ± 1.26	1.03 ± 0.78
		1/4	2.00	0.00
		1/2	2.00	1.00
		3/4	3.00	2.00
	nurse	average	1.63 ± 1.59*	1.00 ± 0.79
		1/4	1.00	0.00
		1/2	2.00	1.00
		3/4	2.00	2.00
Professional title	senior	average	2.33 ± 1.20	1.05 ± 0.79
		1/4	2.00	0.00
		1/2	2.00	1.00
		3/4	3.00	2.00
	intermediate	average	2.57 ± 1.40	0.98 ± 0.80
		1/4	2.00	0.00
		1/2	2.00	1.00
		3/4	4.00	1.75
	junior	average	1.83 ± 1.31	0.96 ± 0.79
		1/4	1.00	0.00
		1/2	2.00	1.00
		3/4	2.25	2.00
Tropics working experience	Yes	average	2.37 ± 1.23	1.01 ± 0.78
		1/4	2.00	0.00
		1/2	2.00	1.00
		3/4	3.00	2.00
	No	average	2.03 ± 1.52	1.13 ± 0.86
		1/4	1.00	0.25
		1/2	2.00	1.00
		3/4	3.00	2.00

**Note**: The total score of each part was 10. *p<0.05: doctor vs. nurse

## Discussion

This study focused on the oral complications related to tropical infectious diseases in tropical regions, but not oral lesions in tropical areas. There were two reasons for this distinction. First, the recognition of oral complications related to tropical infectious diseases might facilitate the early diagnosis of tropical infectious diseases. Secondly, good clinical management of the oral complications related to tropical infectious diseases might improve the control of these infectious diseases. Tropical infectious diseases are often accompanied by a range of oral complications [8, 15]; thus, the clinical management of such oral complications is an indispensable factor in the prevention and control of tropical infectious diseases. It is clear that the awareness of patients with regards to tropical infectious diseases and oral health is insufficient [33]; patients are not able to ensure self-protection and health care. Therefore, the cognition of dental professionals and infectious disease practitioners will directly affect the outcomes of the clinical management and prevention of tropical infectious diseases. According to our previous study, the cognition of dental professionals with regards to tropical infectious diseases and oral health was unsatisfactory. However, only scant research has addressed the understanding of infectious disease practitioners with regards to the association between tropical infectious diseases and oral health.

Based on our investigation, we identified nine key facts that need to be considered in the future. First, it was clear that infectious disease practitioners have not paid enough attention to tropical infectious diseases and oral health; this may be due to the difficulty in identifying the relevant oral complications or ignorance with regards to relevant oral complications. There was no significant impact with regards to the degree of attention between having tropical working experience and not having such experience, thus suggesting that practitioners did not pay specific attention to these issues in different working experiences. Secondly, the attitude of infectious disease practitioners with regards to the necessity and importance of learning about tropical infectious diseases and oral health was highly insufficient; there were no significant differences between practitioners with different backgrounds and experiences. Third, the knowledge of infectious disease practitioners with regards to tropical infectious diseases and oral health was notably inadequate, thus suggesting that the importance of oral manifestations related to tropical infectious diseases has been considered adequately in the clinical management and prevention of tropical infectious diseases. Fourth, education background, professional identity, professional title, and experience of working in the tropics did not affect the levels of appropriate knowledge, thus indicating that the popularization of knowledge was unsatisfactory in terms of academic, clinical and continuing education. Fifth, there was a lack of breadth and depth of cognition among infectious disease practitioners, although some exhibited positive attitudes towards tropical infectious diseases and oral health. Sixth, the lack of such knowledge and cognition could create certain risks in the prevention and control of tropical infectious diseases by infectious disease practitioners. Seventh, we found that the understanding of doctors was better than that of nurses. There may be two reasons for this. First, the popularization of knowledge of the medical specialty in this field was better than that of the nursing specialty. Secondly, doctors may have more direct understanding and feeling of the diagnosis and treatment of relevant diseases in clinical practice. Eighth, compared to our previous study, we found that the understanding of infectious disease practitioners was lower than that of dental professionals [8]. This may be because some oral complications occurred as the initial sign of tropical infectious diseases, and the patients first visited their dental professionals. These experiences might help dentists to learn relevant knowledge. However, these oral manifestations might be neglected by infectious disease practitioners in cases that had multiple serious systemic disorders. Finally, we observed significantly low levels of knowledge; this may be due to two factors. First, infectious disease practitioners did not have a reserve of relevant knowledge; secondly, the questions that featured in the questionnaire might be too difficult for infectious disease practitioners. As shown in Table 3, the attitude of infectious disease practitioners towards tropical infectious diseases and oral health was very unsatisfactory; this may prevent these professionals from learning relevant knowledge. Therefore, the first possibility might be more likely than the second possibility.

Based on the conclusions, we propose several suggestions. First, we should prepare a dedicated handbook to introduce oral complications related to tropical infectious diseases, the oral adverse events caused by the medications used to treat tropical infectious diseases, therapeutic and emergency strategies, the course of disease monitoring, prevention, and control measures. Second, improve the medical education system by offering relevant public courses in college education, academic lectures in continuing education, and relevant clinical practice in clinical education. Third, develop novel practical software to simulate scenarios relating to infections and oral complications. Infectious disease practitioners could use this software to manage the simulation cases by applying a relevant scoring system. Fourth, create a specialized website to share relevant information and provide an efficient communication platform. Fifth, provide special training according to local epidemiology for the practitioners who work in tropical areas, such as medical services for domestic tropical areas, international assistance, and peace-keeping missions. Our aim is to promote these issues issue and improve the management of tropical infectious diseases by applying our recommendations. In addition, we strongly recommend that infectious disease practitioners follow the following guidelines when encountering suspicious cases. First, conduct an oral examination and inquire about the history of relevant oral diseases and generate detailed records. Second, invite dentists for consultation to clarify whether the observed oral diseases are actual complications of infectious diseases or independent oral diseases. Third, propose an optimal management plan by consultation with dentists. Fourth, ensure cases are followed-up in an appropriate manner. Fifth, draft a case report featuring detailed information.

There are some limitations to this study, that need to be considered. First, the questions featured in the questionnaire may have been too difficult for the infectious disease practitioners; this may have created biased outcomes. In future, it will be necessary to generate a gradient of difficulty when setting questions for the questionnaire. Second, the sample size needs to be expanded to cover a wider range of infectious disease practitioners in China. A larger sample size could reduce errors and provide more representative data. Third, future studies should extend to infectious disease practitioners who are based overseas. It is of great significance to collect data from different countries to fully reveal the conditions and mechanisms that link tropical infectious diseases and oral health. Fourth, further details of experience working in the tropics should be collected, such as the duration and location of such experience. With these parameters, we could critically analyze the factors that influence the perception of tropical infectious diseases and oral health among infectious disease practitioners.

## Conclusion

Oral complications are frequently reported to be associated with tropical infectious diseases. An accurate diagnosis and application of an appropriate management strategy is likely to be conducive to the prevention and control of epidemics. In this study, we described oral complications that are known to be related to tropical infectious diseases. Furthermore, we focused on the understanding of infectious disease practitioners with regards to the association between tropical infectious diseases and oral health. We found that these practitioners had a highly unsatisfactory understanding of the association between these two types of diseases. Consequently, it is vital that we improve specialty education practice for infectious disease practitioners.

### Electronic supplementary material

Below is the link to the electronic supplementary material.

Supplementary Material 1

## Data Availability

All data generated or analyzed during this study are included in this published article.
