# Supplementary material for: Oral Complications related to tropical infectious Diseases: an introduction and analysis of survey data
Source: BMC Oral Health. 2023 Oct 24;23:788. doi: 10.1186/s12903-023-03514-w (PMC10599019; doi:10.1186/s12903-023-03514-w)
Supplement: Supplementary file 1 — Supplementary Material 1 [file 12903_2023_3514_MOESM1_ESM.docx]

**Supplementary data**

**Questionnaire on tropical infectious disease and oral health**

**Please provide your individual information:**

1. Gender

1. Male
2. Female

2. Age

3. Educational background

1. Postgraduate
2. Graduate
3. Junior college diploma

4. Professional identity

1. Doctor
2. Nurse

5. Professional title

1. Senior (chief physician, associate chief physician, chief superintendent nurse, associate chief superintendent nurse)
2. Intermediate (attending physician, nurse-in-charge)
3. Junior (resident physician, primary nurse, nurse)

6. Do you have the tropics working experience?

1. Yes
2. No

**Please select the options based on your actual situation:**

7. Have you focused on tropical infectious disease and oral health?

1. Yes
2. No

8. Have you studied on tropical infectious disease and oral health systematically?

1. Yes
2. No

9. If working in non-tropics, it is necessary to learn about infectious diseases and oral health systematically.

1. Yes
2. No

10. If working in tropics, it is necessary to learn about infectious diseases and oral health systematically.

1. Yes
2. No

11. Do you think the identification of oral lesions is helpful to the management of tropical infectious disease?

1. Yes
2. No

**Please select the options based on your knowledge, each question might have one or more correct options. If you do not know the answer to the question, you can select‘Don’t know’.**

12. Which tropical infectious disease might be accompanied with oral complications?

1. Parasitic diseases
2. Bacterial diseases
3. Viral diseases
4. Fungal diseases
5. Don't know

13. Which tropical infectious disease might be accompanied with oral candidiasis?

1. AIDS
2. Malaria
3. Dengue fever
4. Leprosy
5. Don't know

14. Which tropical infectious disease might be accompanied with oral hairy leukoplakia?

1. AIDS
2. Trypanosomiasis
3. Cholera
4. Malaria
5. Don't know

15. Which tropical infectious disease might be accompanied with oral hemorrhage?

1. Taeniasis
2. Malaria
3. Dengue fever
4. Echinococcosis
5. Don't know

16. Which tropical infectious disease might be accompanied with osteonecrosis of the jaw?

1. Leishmaniasis
2. Malaria
3. Dengue fever
4. Cysticercosis
5. Don't know

17. Which tropical infectious disease might be accompanied with herpes labialis?

1. Malaria
2. Amoebasis
3. Ancylostomiasis
4. Yaws
5. Don't know

18. Which tropical infectious disease might be accompanied with Exuberant granulomatous lesions

1. Measles
2. Rhinosporidiosis
3. Filariasis
4. Leprosy
5. Don't know

19. Which tropical infectious disease might be prone to secondary burkitt lymphoma of the jaw?

1. Dengue fever
2. Taeniasis
3. Malaria
4. Trichuriasis
5. Don't know

20. Which tropical infectious disease might be accompanied with oral depigmentation

1. Leprosy
2. Yaws
3. Malaria
4. Cholera
5. Don't know

21. Which tropical infectious disease might be cause enamel hypoplasias of the children?

1. Cysticercosis
2. Leishmaniasis
3. Echinococcosis
4. Malaria
5. Don't know

22. What are the common oral complications of leishmaniasis？

1. Oral pain
2. Destructive granulomatous lesions of oral mucosa
3. Lipochagomata genii
4. Pericoronitis
5. Don't know

23. What are the common oral complications of Trichuriasis?

1. Ulcerative stomatitis
2. Hyperplastic gingivitis
3. Cystic nodules
4. Glossitis
5. Don't know

24. What are the common oral complications of chronic trypanosomiasis?

1. Myoclonus of lips
2. Gangosa
3. Lipochagomata genii
4. Osteonecrosis of the jaw
5. Don't know

25. Which oral complications of the tropical infectious disease has the high incidence in female patients?

1. Taeniasis
2. Filariasis
3. Yaws
4. Malaria
5. Don't know

26. What is the characteristic oral complication of measles?

1. Koplik's spots on oral mucosa
2. Gangosa
3. Lipochagomata genii
4. Oral lichenoid reaction
5. Don't know

27. What is the characteristic oral complication of yaws?

1. Periodontitis
2. Post-extraction hemorrhage
3. Loosening, dysplasia and dental pulp necrosis of maxillary anterior teeth
4. Gangosa
5. Don't know

28. What is the characteristic oral complication of paracoccidioidomycosis?

1. Painful proliferative erythematous granulomata of oral mucosa
2. Cystic nodules on the oral mucosa
3. Oral lichenoid reaction
4. Loosening, dysplasia and dental pulp necrosis of maxillary anterior teeth
5. Don't know

29. What are the common oral complications of acute dengue fever?

1. Acute oral hemorrhage
2. Erythematous plaque of oral mucosa
3. Oral candidiasis
4. Osteonecrosis of the jaw
5. Don't know

30. What are the common oral complications of chronic malaria?

1. Alveolar bone resorption
2. Burkitt lymphoma of the jaw
3. Enamel hypoplasias
4. Oral pigment
5. Don't know

31. Which antimalarials might have oral side effects?

1. Artemisinin and its derivatives
2. Chloroquine
3. Amodiaquine
4. Sulfadoxine–pyrimethamine
5. Don't know

32. What are the oral side effects of antimalarials?

1. Enamel hypoplasia
2. Tooth discoloration
3. Oral lichenoid reaction
4. Salivary gland disorders
5. Don't know

33. What is the key for infectious disease practitioner to prevent infection when conduct oral examinations in the patients with malaria or dengue fever?

1. Avoid contact with the patient's blood or body fluids
2. Mosquito control
3. Fleas and flies control
4. Clean the water supply and drainage system of the consulting room
5. Don't know

34. What is the key for infectious disease practitioner to prevent infection when conduct oral examinations in the patients with amoebasis or trichinosis?

1. Avoid contact with the patient's blood or body fluids
2. Mosquito control
3. Fleas and flies control
4. Clean the water supply and drainage system of the consulting room
5. Don't know

35. What are the commonly used treatments for acute oral hemorrhage?

1. Hemostasis by compression
2. Hemostasis by suture
3. Hemostatic drugs administration
4. Lying flat and rest
5. Don't know

36. What are the commonly used treatments for oral candidiasis?

1. 2%-4% sodium bicarbonate solution
2. 0.2% Chlorhexidine solution
3. Fluconazole
4. Surgery
5. Don't know
